# Descriptive Psychopathology of the Acute Effects of Intravenous Delta-9-Tetrahydrocannabinol Administration in Humans

**DOI:** 10.3390/brainsci9040093

**Published:** 2019-04-25

**Authors:** Marco Colizzi, Nathalie Weltens, Philip McGuire, Lukas Van Oudenhove, Sagnik Bhattacharyya

**Affiliations:** 1 Department of Psychosis Studies, Institute of Psychiatry, Psychology and Neuroscience, King’s College London, London SE5 8AF, UK; philip.mcguire@kcl.ac.uk (P.M.); sagnik.2.bhattacharyya@kcl.ac.uk (S.B.); 2 Translational Research Center for Gastrointestinal Disorders (TARGID), Department of Chronic Diseases, Metabolism and Ageing, University of Leuven, Leuven 3000, Belgium; nathalie.weltens@kuleuven.be (N.W.); lukas.vanoudenhove@kuleuven.be (L.V.O.)

**Keywords:** delta-9-tetrahydrocannabinol, placebo, cannabis-associated psychosis, schizophrenia

## Abstract

Background: Cannabis use can increase the risk of psychosis, and the acute administration of its key psychoactive ingredient, delta-9-tetrahydrocannabinol (∆9-THC), can induce transient psychotomimetic symptoms. Methods: A double-blind, randomized, placebo-controlled crossover design was used to investigate the symptomatic effects of acute intravenous administration of ∆9-THC (1.19 mg/2 mL) in 16 healthy participants (seven males) with modest previous cannabis exposure. Results: In the 20 min following acute ∆9-THC administration, symptomatic effects of at least mild severity were present in 94% of the cohort, with moderate to severe symptoms having a much lower prevalence (19%). Nearly one-third (31%) of the volunteers were still experiencing protracted mild symptomatic effects 2.5 h after exposure to ∆9-THC. Compared to the Δ9-THC challenge, most of the study participants did not experience any symptomatic effects following placebo administration (62%). Acute physical reactions were 2.5 times more frequent after Δ9-THC (31%) than placebo (12%). Male and female participants differed in terms of acute Δ9-THC effects, with some negative symptoms occurring more frequently in female (56% to 89%) than male participants (0% to 29%), and acute physical reactions occurring exclusively in the female gender (56%). Conclusions: These results have implications for future research, also in light of cannabis being the most widely used illicit drug.

## 1. Introduction

Psychosis is a severe mental disorder resulting from a complex interplay between genetic and environmental determinants leading to a disruption of central nervous system function [1]. In order to better understand its pathophysiological mechanisms, different models of psychosis have been proposed [2]. Over the last two decades, there has been growing interest in the drug-induced model of psychosis, due to the potential of several pharmacological agents to elicit psychotomimetic symptoms that resemble those observed in psychosis patients [3]. In particular, in-human models of psychosis have become available involving the acute administration of dopaminergic [4], serotoninergic [5], glutamatergic [6], and cannabinoid compounds [7,8]. Compared to animal models, which have been implicated as not adequately modeling the complexity of the disorder [9], the transient symptoms induced by acute challenge with psychotomimetic drugs in healthy individuals are of interest, as they may share pathophysiological mechanisms with the full-blown disorder.

The administration of cannabis’ key psychoactive ingredient delta-9-tetrahydrocannabinol (∆9-THC) has been shown to induce transient psychosis-like symptoms in otherwise healthy individuals [10,11,12,13]. The association between cannabinoids and psychosis is further supported by several lines of research: (i) the evidence for a higher risk of psychosis in cannabis users [14,15,16], especially against a specific genetic background [17,18]; (ii) the evidence that cannabis use can exacerbate psychotic symptoms and cause relapse in patients with schizophrenia [19,20,21,22,23]; and (iii) the evidence that the endocannabinoid system might be disrupted in patients with schizophrenia both in the context of cannabis use and in its absence [24,25], as well as involved in modulating cognitive function in healthy individuals [26,27,28]. 

Although clinical research is needed to further understand psychosis in cannabis users, limited evidence from anecdotal studies has been published on the nature of the transient clinical manifestations of acute cannabis intoxication in healthy individuals [29,30,31]. In many respects, experimental studies examining the nature of the psychotomimetic effects of ∆9-THC may arguably be a priority because they can inform further studies of cannabis-associated psychosis, including aetiology, course, prognosis, and treatment. Previous studies that have assessed the acute psychotomimetic effects of ∆9-THC have reported them as summary measure using the PANSS (Positive and Negative Syndrome Scale) [11,12,32,33,34,35,36], BPRS (Brief Psychiatric Rating Scale) [37], SSPS (State Social Paranoia Scale) [35], or self-report questionnaires [12,32,34]. A limited range of other effects has also been investigated using self-report questionnaires and visual analogue measures, including dissociation [12], affect and mood [11,12,32,34,35,36,37], sedation and intoxication [11,12,36,37], and anxiety and panic [11,12,36].

Also, evidence indicates that frequent cannabis users have a more blunted response to the acute psychotomimetic effects of ∆9-THC compared to a group of healthy controls, suggesting the potential development of tolerance [38,39]. Thus, studies conducted among frequent users may have limited usefulness in informing on the nature of the symptoms acutely induced by cannabis in healthy individuals. 

Employing a placebo-controlled acute pharmacological challenge design, the aim of this study was to investigate the symptomatic effects of acute ∆9-THC administration under controlled experimental conditions in a group of healthy individuals with modest previous cannabis use.

## 2. Materials and Methods

This study employed a double-blind, randomized, placebo-controlled, repeated-measures, within-subject design, with a counterbalanced order of drug administration, using an established protocol [13,40]. Sixteen healthy participants (seven males) were assessed on two different occasions separated by at least a two-week interval, with each session preceded by intravenous administration of Δ9-THC (1.19 mg/ 2 mL) or placebo. All the subjects underwent structural Magnetic Resonance Imaging (MRI), functional MRI (fMRI) and proton magnetic resonance spectroscopy (1H-MRS) scanning in both sessions. The present report focuses on the psychopathological assessment.

### 2.1. Experimental Procedure 

Prior to each study visit, participants were advised to get at least six to eight hours sleep overnight and to refrain from smoking for four hours, taking caffeine for 12 h, and consuming alcohol for 24 h. Also, subjects had been abstinent from cannabis for at least six months before the first study visit, and were advised to abstain from using any substance throughout the duration of the study. On arrival at the study center in the morning, participants had a light standardized breakfast after an overnight fast. All the subjects had a negative urinary drug screen for amphetamines, benzodiazepines, cocaine, opiates, and Δ9-THC, and were tested on each study day using immunometric assay kits. All the female participants had a negative pregnancy test; also, all of them were consistently using a reliable contraceptive method, apart from a single subject who underwent both study visits in the first week of the menstrual cycle. After a physical examination performed by a medical doctor, an indwelling intravenous line in the non-dominant arm was placed by a trained nurse. This cannula was used for the intravenous administration of Δ9-THC (1.19 mg/ 2 mL, ≥99% pure; THC-Pharm, Frankfurt, Germany, http://biochem.thc-pharm.de; pharmaceutical formulation at the Barts Health NHS Trust pharmacy according to previous work [41]) or placebo as well as blood collection a different time points before and after drug challenge. A dose of 1.19 mg was used, as previous work has suggested that an intravenous dose range between 0.015–0.03 mg/kg is consistently associated with an induction of psychotomimetic symptoms [42]. Heart rate and blood pressure were monitored via a digital recorder and an automated arm cuff for the entire duration of the study. 

### 2.2. Subjects

Sixteen healthy, English-speaking, right-handed individuals participated in this study. Demographic information such as age, ethnicity, and level of education was recorded. All the subjects gave written, informed consent, and completed all of the components of the study. Personal or family history of psychiatric illness in first-degree relatives represented an exclusion criterion. None of the subjects included in the study had used more than 21 units/week of alcohol on a regular basis. Only three subjects had a regular smoking habit (two of them smoking <10 cigarettes/day and one smoking two cigarettes/week), six had smoked occasionally/experimentally, and seven had never smoked. Apart from three subjects who had a single experimental use of 3,4-Methylenedioxymethamphetamine (MDMA), all the remaining participants had never used any other substance. Regarding previous lifetime cannabis exposure, nine subjects had used cannabis ≤5 times, three subjects ≤10 times, two subjects ≤20 times, one subject 20 times, and one subject 60 times.

### 2.3. Psychopathological Assessment

All the participants were interviewed by a psychiatrist with a specific expertise in Diagnostic and Statistical Manual of Mental Disorders, 5th edition (DSM-5) schizophrenia and other psychotic disorders as well as substance use disorders [43], using the Structured Clinical Interview for DSM-5 (SCID-5) as a guide for the assessment of the psychotic spectrum [44]. Assessments were carried out immediately before and at 20 min and 2.5 h after drug administration, and clinically discussed with a senior psychiatrist at the end of each study visit. Psychopathological ratings were recorded using the Positive and Negative Syndrome Scale [45] (PANSS), which is a well-established scale that is used for measuring the symptom severity of individuals with psychosis. Verbatim quotations from participants were also recorded, as research evidence indicates that the inclusion of excerpts from transcripts might help clarify links between data, interpretation, and conclusion [46]. Participants were contacted the day after each study visit for a health check as part of the study standard operating procedure (SOP). Putative symptoms lasting longer than expected or occurring after the end of the study visit were also recorded.

### 2.4. Ethics Approval

The study was approved by the Joint South London and Maudsley (SLaM) and Institute of Psychiatry, Psychology & Neuroscience (IoPPN) National Health Service Research Ethics Committee (PNM/13/14-38), and the investigators had a license to use Δ9-THC for research purposes.

## 3. Results

### 3.1. Demographic Information

Study participants had a mean age of 24.44 (standard deviation, SD: 4.29) years. All except three (with self-described mixed ethnic origin) of the volunteers were white Europeans. They had 16.94 ± 2.84 years (mean, M ± SD) of education. 

The effects of ∆9-THC administration on blood pressure and heart rate and related statistics as well as Δ9-THC plasma levels have been previously reported [40].

### 3.2. Prevalence and Severity of Symptoms: Results at a Glance

#### 3.2.1. Following Acute ∆9-THC Administration

Apart from one participant with minimal and questionable symptoms, who did not score more than two on any PANSS item, the entire study cohort reported at least mild and clearly detectable symptomatic effects (94%; ≥3 on at least one PANSS item) within 20 min following acute challenge with Δ9-THC. More severe symptomatic effects were experienced by a smaller proportion of participants, with 10 volunteers reporting at least moderate symptoms (62%; ≥4 on at least one PANSS item) and three of them reporting moderate to severe symptoms (19%; ≥5 on at least one PANSS item). Acute physical reactions, including effects on movement, blood pressure, heart rate, skin vascularity, and vagal response, occurred on five occasions (31%).

Two hours and 30 minutes after the intravenous administration of Δ9-THC, five (31%) and three (19%) participants were still experiencing mild (= 3 on at least one PANSS item) and minimal (= 2 on at least one PANSS item) symptoms, respectively. In contrast, by this time, no physical reaction was evident. Upon telephone follow-up, six participants (37%) reported long-lasting effects of the drug, which faded away by the end of the study day or the subsequent morning. These symptoms were mainly included fatigue and food craving. In one case, these effects included psychosis-related symptoms such as suspiciousness, hostility, tension, and poor impulse control (6%).

#### 3.2.2. Following Placebo Administration

Differently from the Δ9-THC condition, most of the study participants did not experience any symptomatic effects following placebo administration (*n* = 10; 62%). Three volunteers (19%) reported minimal and questionable symptoms (= 2 on at least one PANSS item) and, interestingly, only three subjects (19%) had detectable symptoms of mild severity (= 3 on at least one PANSS item). Acute physical reactions, including effects on heart rate and skin vascularity, were present in two occasions (12%), occurring at a lower rate compared to the Δ9-THC condition.

Two hours and 30 minutes after the intravenous administration of placebo, only one participant (6%) was experiencing minimal and questionable psychotomimetic symptoms (= 2 on at least one PANSS item). Also, similarly to the Δ9-THC condition, no physical reaction was evident at that time point. Finally, differently from the Δ9-THC condition, only one participant (6%) reported long-lasting effects at the telephone follow-up after placebo administration, which faded away by the end of the day. However, these effects included psychosis-related symptoms such as suspiciousness, which was totally overlapping with the frequency of long-lasting psychosis-related symptoms following acute challenge with Δ9-THC (6%).

### 3.3. Symptoms Description

#### 3.3.1. Psychosis-Related Positive Symptoms and Disorganization

The effects of ∆9-THC administration on the PANSS positive symptom subscale and related statistics have been previously reported [40].

Conceptual disorganization was the most frequently observed symptom in the ~20 min following the acute administration of Δ9-THC, with all the participants reporting such symptoms in a minimal to severe form (2 ≤ PANSS-related item ≤ 6). Further frequent symptoms (≥2 on PANSS-related item) included hallucinatory behavior (62%), excitement (62%), and suspiciousness/persecution (56%). A lower percentage of participants also reported symptoms of grandiosity (25%), hostility (19%), and delusions (19%).

Some symptoms were still detectable 2.5 h after the injection (≥2 on PANSS-related item), even if in a more attenuated form, with conceptual disorganization being the most frequent symptom (37%), followed by hallucinatory behavior (6%) and excitement (6%). Volunteers showing a more severe conceptual disorganization immediately after the intravenous administration of Δ9-THC were more likely to still experience such symptom 2.5 h after the injection, with five out of nine participants experiencing moderate to severe conceptual disorganization (4 ≤ PANSS-related item ≤ 6) versus one out of seven participants with minimal to mild conceptual disorganization (≤3 on PANSS-related item).

In the ~20 min following the acute administration of placebo, positive symptoms were reported by four participants (≥2 on PANSS-related item) and only two of them had clearly detectable symptoms (mild severity, ≥3 on PANSS-related item; 12%), which was a percentage that was 7.5 times smaller than that observed in participants under the influence of Δ9-THC. In both cases, these symptoms were within the conceptual disorganization domain. Also, only one participant was still experiencing a disorganized process of thinking 2.5 h after the injection of placebo.

Overall, Δ9-THC-induced excitement and grandiosity were more frequent in male (86% and 43% respectively) than female participants (44% and 11% respectively). Instead, hostility was observable only in a percentage of female participants (33%) (Table 1).

#### 3.3.2. Psychosis-Related Negative Symptoms

The effects of ∆9-THC administration on the PANSS negative symptom subscale and related statistics have been previously reported [40].

A lack of spontaneity and reduced flow of conversation was the most frequently observed symptom in the ~20 min following the acute administration of Δ9-THC, with 13 participants reporting such symptoms in a minimal to moderately severe form (2 ≤ PANSS-related item ≤ 5; 81%). Further frequent symptoms (≥2 on PANSS-related item) included stereotyped thinking (69%), blunted affect (62%), poor rapport (62%), and difficulty in abstract thinking (50%). A lower percentage of participants also reported emotional (44%) and social withdrawal (31%). In only three participants (19%), some symptoms were still detectable 2.5 h after the injection, even if in a more attenuated form (lack of spontaneity and reduced flow of conversation, 12%; blunted affect, 6%; difficulty in abstract thinking, 6%; stereotyped thinking, 6%). 

In the ~20 min following the acute administration of placebo, negative symptoms were detectable only in two participants (mild severity, = 3 on PANSS-related item, 12%), which was a percentage that was 7.5 times smaller than that observed in participants under the influence of Δ9-THC. In these cases, symptoms included the poor rapport (6%) and/or the lack of spontaneity (12%) domains. Also, only one participant was still experiencing a lack of spontaneity and reduced flow of conversation 2.5 h after the injection of placebo.

Overall, Δ9-THC-induced poor rapport, emotional withdrawal, and social withdrawal were more frequent in female participants (56% to 89%) than male participants (0% to 29%) (Table 2).

#### 3.3.3. Psychosis-Related General Psychopathology

The effects of ∆9-THC administration on the PANSS general psychopathology subscale and related statistics have been previously reported [40].

Poor attention was the most frequently observed symptom in the ~20 min following the acute administration of Δ9-THC, with 14 participants reporting such symptoms in a mild to moderate form (3 ≤ PANSS-related item ≤ 4; 87%). Most of the participants also experienced a disturbance of volition (75%), disorientation (69%), and poor impulse control (62%). Further frequent symptoms (≥ 2 on PANSS-related item) included somatic concern (50%), preoccupation (50%), motor retardation (50%), mannerisms and posturing (50%), unusual thought content (50%), tension (44%), and active social avoidance (44%). A lower percentage of participants also reported a lack of judgment and insight (37%), symptoms of anxiety and depression (31%), uncooperativeness (31%), and feelings of guilt (12%). One participant reported feeling less depressed after the acute challenge with Δ9-THC.

Some symptoms were still detectable 2.5 h after the injection (≥2 on PANSS-related item), even if in a more attenuated form, with poor attention and disorientation being the most frequent symptom (31%), followed by motor retardation (12%), somatic concern (6%), preoccupation (6%), anxiety (6%), tension (6%), and uncooperativeness (6%). 

In the ~20 min following the acute administration of placebo, general psychopathological symptoms were observable in six participants (≥2 on PANSS-related item) and only three of them had clearly detectable symptoms (mild severity, = 3 on PANSS-related item; 19%), which was a percentage that was five times smaller than that observed in participants under the influence of Δ9-THC. In these cases, symptoms included poor attention (12%), somatic concern and preoccupation (6%), tension (6%), disorientation (6%), disturbance of volition (6%), and poor impulse control (6%). Also, only one participant was still experiencing a disturbance of volition 2.5 h after the injection of placebo. Male and female participants were similar in terms of the prevalence of Δ9-THC-induced general psychopathology (Table 3).

### 3.4. Subjects’ Quotes

Table 4 reports the most relevant symptoms experienced by the participants from a narrative perspective. The quality of symptoms showed similarity to the psychotic symptoms reported by schizophrenia patients. When under the acute effect of Δ9-THC, individuals reported both positive and negative symptoms. The most relevant positive symptoms induced by Δ9-THC included suspiciousness, paranoid and grandiose ideas/delusions, conceptual disorganization, and perceptual alterations. Negative symptoms included reduced rapport, a lack of spontaneity, emotional withdrawal, and concrete thinking. The administration of Δ9-THC also induced altered body perception, depersonalization/derealization, slowing of time, euphoria, and anxiety.

### 3.5. Additional Symptoms

Additional symptoms not necessarily related to psychosis also occurred during the trial. In particular, five female participants (56%) had an acute physical reaction to the Δ9-THC administration, including generalized tremors, vagal reaction, paleness, orthostatic hypotension, sick feeling, flushing, and symptoms of fainting. In contrast, no male participant experienced any physical reaction after the drug challenge. Less intense physical reactions also occurred during the placebo session in two occasions (Table 5).

**Figure 1:**
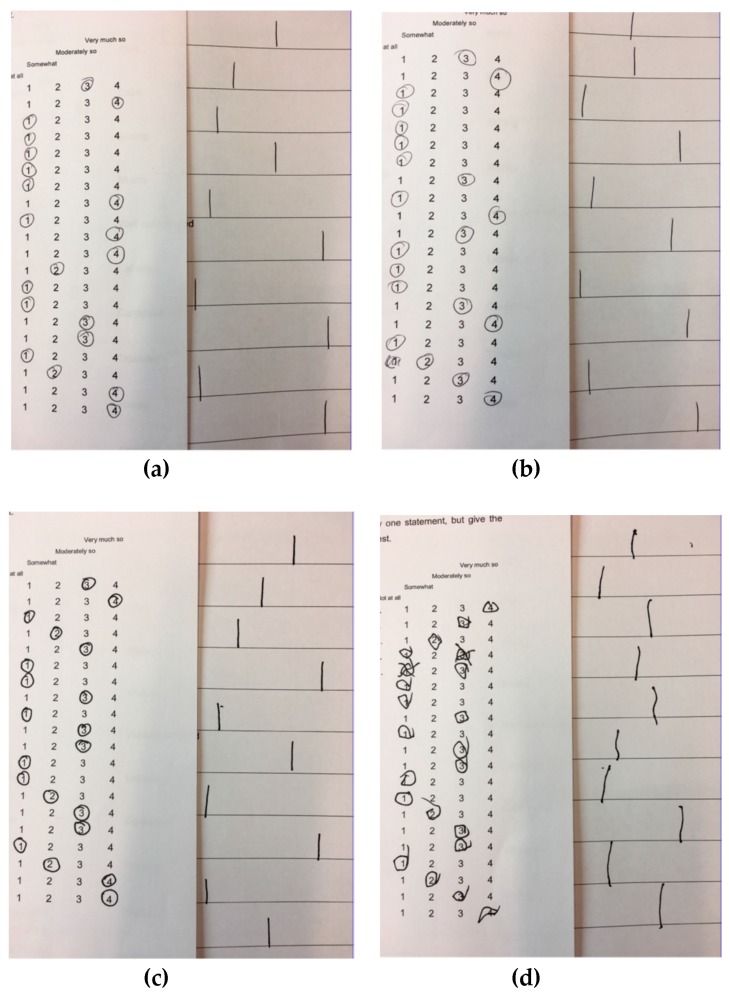
Difficulty in motor coordination and indecisiveness. (**a**) Before placebo administration; (**b**) After placebo administration; (**c**) Before delta-9-tetrahydrocannabinol administration; (**d**) After delta-9-tetrahydrocannabinol administration.

After the intravenous administration of Δ9-THC, eight volunteers (50%) showed difficulty in motor coordination and indecisiveness with different levels of severity (Table 5; the most severe occurrence is shown in Figure 1). A participant had a protracted posture alteration. Also, two participants showed over-inclusive thinking and protracted internal absorption, respectively (Table 5).

## 4. Discussion

The purpose of this clinical investigation was to systematically assess the transient psychotic reaction to the intravenous administration of pure Δ9-THC in healthy subjects in a controlled setting, which was in line with the evidence that this cannabinoid represents a valid pharmacologic model for psychosis [10,11,12]. Results from this study indicate: (i) detectable acute Δ9-THC-induced symptomatic effects in over 90% of the cohort, with moderate to severe symptoms having a lower prevalence (<20%); (ii) protracted minimal to mild Δ9-THC-induced symptomatic effects in 50% of the cohort (~2.5 h after the exposure); (iii) acute physical reactions to Δ9-THC in about 30% of the cohort and only in female participants; (iv) long-lasting Δ9-THC-induced physical symptoms and psychosis-related symptomatic effects in less than 40% and 6% of the cohort, respectively; (v) detectable and mild symptomatic effects after placebo administration in less than 20% of the cohort; (vi) protracted minimal and questionable symptomatic effects after placebo administration in 6% of the cohort (~2.5 h after the exposure); (vii) acute physical reactions to placebo in about 12% of the cohort and only in female participants; and (viii) long-lasting symptomatic effects of placebo in only 6% of the cohort.

The constellation of symptomatic effects induced by Δ9-THC resembled several dimensions of psychotic disorders and overlapped with evidence from previous acute challenge studies with Δ9-THC [12]. However, in order to better understand the extent of its detrimental effects, this investigation took into account the potential nonspecific effects of the drug administration, the so-called placebo effects when they are beneficial, and nocebo effects when they are harmful [47]. Study participants reported a number of symptoms and signs when administered placebo, indicating a nocebo effect. Both psychological (conditioning, negative expectations) and neurobiological (cholecystokinin, endogenous opioids, and dopamine) mechanisms might explain the nocebo effects observed in this study [48]. When controlling for the prevalence, quality, and severity of the subjective and objective changes occurring under placebo, the manifestation of symptomatic effects following Δ9-THC administration remained significantly higher and of greater severity, with most of the transient psychosis-like symptoms occurring only under Δ9-THC. Also, psychotomimetic symptoms lasted significantly longer under Δ9-THC compared to the placebo condition. Similarly, some objective protracted symptoms such as poor motor coordination, posture alteration, over-inclusive thinking, and internal absorption occurred only under Δ9-THC.

Relatively longer-lasting (<24 h) self-reported effects such as tiredness, sleepiness, and increased appetite occurred only under Δ9-THC. Acute physical reactions to the intravenous administration of the drug were more prevalent and clinically more severe in participants under the influence of Δ9-THC than under placebo. Also, they appeared to be gender-specific, with only female participants showing such reactions. Physical and somatic effects were not unexpected, as Δ9-THC has been shown to acutely induce sedation and intoxication [40]. 

Upon comparing results from this study with previous research, several factors need to be considered, including, but not limited to, the degree of current cannabis use (tolerance effect) and lifetime cannabis exposure (residual effect) of the study samples, and study design. Some research evidence indicates less prominent acute behavioral effects of Δ9-THC in current cannabis users [49], individuals with a past history of frequent cannabis use [38], and when administering Δ9-THC orally [50], as also recently reviewed [39]. Further evidence suggests that the development of tolerance may be explained by the less marked effects of acute Δ9-THC administration on brain function [51,52]. Participants taking part in our intravenous Δ9-THC challenge had been abstinent from cannabis for at least six months. Apart from one subject, the study cohort had also modest previous cannabis exposure. Altogether, previous evidence and our findings suggest that healthy subjects with modest previous cannabis exposure and a proper abstinent period might be more reliable to study the psychotropic effect of Δ9-THC and its underlying mechanisms.

Only individuals with negligible use of other substances (alcohol, tobacco, and other illicit drugs) were invited to take part in the study. Therefore, we can reasonably rule out the possibility that some of the results observed could be attributed to the effects of other substance use. Moreover, this study observed an interval between the two study visits of at least 14 days. This allows us to exclude the possibility of carryover effects from the first session, in light of evidence that Δ9-THC has an elimination half-life of 18 h to 4.3 days [53]. Furthermore, all the participants’ urine samples collected at each study visit baseline were negative for the presence of Δ9-THC.

For the purpose of the study and due to ethical reasons, individuals with cannabis dependence or previous negative response to cannabis were excluded from the study. While this allowed us to examine a more homogeneous sample, this might have limited the application of the present results to the general population. Also, caution should be used when making inferences to the general population, as this experiment was conducted in a relatively small sample. The intravenous route of administration was used to allow much more consistent Δ9-THC blood levels across participants and potentially reduce inter-individual variability in drug response [12]. For instance, absorption is slower when cannabinoids are ingested, with Δ9-THC peak concentrations that are lower and more delayed [54], and absorption may also considerably vary between subjects [55]. Similarly, another line of research suggests that cannabinoid levels following cannabis smoking may vary depending on how intensively and efficiently people smoke [56]. However, the intravenous route of administration might have affected the generalizability of the results to the effects of recreational cannabis use. Future studies are needed to assess in the same individuals the effects of acute cannabis challenge using different routes of administration.

## 5. Conclusions

In conclusion, these results provide further evidence of the psychoactive properties of Δ9-THC and have implications for research in this area. Acute psychosis can be secondary to cannabis use, with some patients relapsing with a similar presentation, and a proportion developing a long-lasting psychotic disorder [57]. More research is needed into the chronology and components of the onset of cannabis-associated psychosis. Acute Δ9-THC challenge studies may help elucidate the nature of psychotic symptom development in cannabis users, ultimately enhancing our understanding of the onset, course, and outcome of cannabis-associated psychosis.

## Figures and Tables

**Table 1:** Psychosis-related positive symptoms and disorganization.

Study Participant	Drug	Delusions	Conceptual Disorganization	Hallucinatory Behavior	Excitement	Grandiosity	Suspiciousness/Persecution	Hostility
**male 1**	Δ9-THC	✕	**✓ mild**	✕	✓ minimal	✕	✓ mild	✕
**male 2**	Δ9-THC	✕	✓ mild	✕	✓ mild	✓ minimal	✕	✕
**male 3**	Δ9-THC	✕	**✓ severe**	✓ mild	**✓ moderate**	✓ moderate	✓ mild	✕
**male 4**	Δ9-THC	✕	✓ minimal	✕	✓ minimal	✕	✕	✕
**male 5**	Δ9-THC	✕	✓ moderate	✓ mild	✕	✕	✓ minimal	✕
**male 6**	Δ9-THC	✓ mild	✓ moderate	✓ mild	✓ moderate	✓ moderate	✕	✕
**male 7**	Δ9-THC	✕	**✓ moderate**	**✓ mild**	✓ mild	✕	✓ minimal	✕
**female 1**	Δ9-THC	✕	✓ mild	✕	✕	✕	✕	✓ mild
**female 2**	Δ9-THC	✕	✓ mild	✓ mild	✕	✕	✕	✕
**female 3**	Δ9-THC	✕	✓ mild	✕	✓ mild	✕	✕	✕
**female 4**	Δ9-THC	✕	**✓ moderate**	✓ minimal	✓ mild	✕	✓ mild	✓ mild
**female 5**	Δ9-THC	✕	✓ mild	✓ mild	✓ mild	✕	✓ mild	✕
**female 6**	Δ9-THC	✕	**✓ moderate**	✓ mild	✓ minimal	✕	✕	✕
**female 7**	Δ9-THC	✕	✓ moderate	✕	✕	✕	✓ minimal	✕
**female 8**	Δ9-THC	✓ moderate	**✓ moderate/severe**	✓ severe	✕	✓ moderate	✓ minimal	✕
**female 9**	Δ9-THC	✓ minimal	✓ moderate	✓ moderate	✕	✕	✓ moderate	✓ mild
**male 1**	placebo	✕	✕	✕	✕	✕	✕	✕
**male 2**	placebo	✕	✕	✕	✕	✕	✕	✕
**male 3**	placebo	✕	✕	✕	✓ minimal	✕	✕	✕
**male 4**	placebo	✕	✕	✕	✕	✕	✕	✕
**male 5**	placebo	✕	✓ mild	✕	✕	✕	✕	✕
**male 6**	placebo	✕	✕	✕	✕	✕	✕	✕
**male 7**	placebo	✕	**✓ mild**	**✓ minimal**	✕	✕	✕	✕
**female 1**	placebo	✕	✕	✕	✕	✕	✕	✕
**female 2**	placebo	✕	✕	✕	✕	✕	✕	✕
**female 3**	placebo	✕	✕	✕	✓ minimal	✕	✕	✕
**female 4**	placebo	✕	✕	✕	✕	✕	✕	✕
**female 5**	placebo	✕	✕	✕	✕	✕	✕	✕
**female 6**	placebo	✕	✕	✕	✕	✕	✕	✕
**female 7**	placebo	✕	✕	✕	✕	✕	✕	✕
**female 8**	placebo	✕	✕	✕	✕	✕	✕	✕
**female 9**	placebo	✕	✕	✕	✕	✕	✕	✕

Δ9-THC, (−)-trans-Δ9-tetrahydrocannabinol; symptoms highlighted in bold were still observable 2.5 h after the injection.

**Table 2:** Psychosis-related negative symptoms.

Study Participant	Drug	Blunted Affect	Emotional Withdrawal	Poor Rapport	Social Withdrawal	Difficulty in Abstract Thinking	Lack of Spontaneity and Flow of Conversation	Stereotyped Thinking
**male 1**	Δ9-THC	**✓ minimal**	✕	✕	✕	✕	**✓ mild**	**✓ minimal**
**male 2**	Δ9-THC	✓ mild	✕	✕	✕	✓ mild	✓ mild	✓ minimal
**male 3**	Δ9-THC	✕	✕	✓ mild	✕	**✓ moderate**	✓ minimal	✓ mild
**male 4**	Δ9-THC	✕	✕	✕	✕	✕	✕	✕
**male 5**	Δ9-THC	✕	✕	✕	✕	✕	✓ moderate	✓ minimal
**male 6**	Δ9-THC	✕	✕	✕	✕	✓ mild	✕	✓ minimal
**male 7**	Δ9-THC	✓ mild	✕	✓ moderate	✕	✕	✓ moderate	✓ mild
**female 1**	Δ9-THC	✓ minimal	✓ mild	✓ mild	✓ mild	✓ mild	✓ mild	✓ mild
**female 2**	Δ9-THC	✕	✕	✕	✕	✕	✓ mild	✕
**female 3**	Δ9-THC	✕	✓ mild	✓ minimal	✕	✕	✓ mild	✕
**female 4**	Δ9-THC	✓ mild	✓ mild	✓ mild	✕	✓ moderate	✓ moderate	✓ mild
**female 5**	Δ9-THC	✓ mild	✕	✓ mild	✓ mild	✓ moderate	✓ mild	✓ mild
**female 6**	Δ9-THC	✓ moderate	✓ mild	✓ mild	✓ mild	✕	✓ moderate	✕
**female 7**	Δ9-THC	✓ mild	✓ moderate	✓ mild	✓ mild	✓ moderate/ severe	✓ mild	✕
**female 8**	Δ9-THC	✓ moderate/severe	✓ moderate/severe	✓ severe	✓ moderate	✓ moderate	**✓ moderate/severe**	✓ moderate
**female 9**	Δ9-THC	✓ mild	✓ minimal	✓ mild	✕	✕	✕	✓ minimal
**male 1**	placebo	✕	✕	✕	✕	✕	✕	✕
**male 2**	placebo	✕	✕	✕	✕	✕	✕	✕
**male 3**	placebo	✕	✕	✕	✕	✕	✕	✕
**male 4**	placebo	✕	✕	✕	✕	✕	✕	✕
**male 5**	placebo	✕	✕	✕	✕	✕	✓ mild	✕
**male 6**	placebo	✕	✕	✕	✕	✕	✕	✕
**male 7**	placebo	✕	✕	✓ mild	✕	✕	**✓ mild**	✕
**female 1**	placebo	✕	✕	✕	✕	✕	✕	✕
**female 2**	placebo	✕	✕	✕	✕	✕	✕	✕
**female 3**	placebo	✕	✕	✕	✕	✕	✕	✕
**female 4**	placebo	✕	✕	✕	✕	✕	✕	✕
**female 5**	placebo	✕	✕	✕	✕	✕	✕	✕
**female 6**	placebo	✕	✕	✕	✕	✕	✕	✕
**female 7**	placebo	✕	✕	✕	✕	✕	✕	✕
**female 8**	placebo	✕	✕	✕	✕	✕	✕	✕
**female 9**	placebo	✕	✕	✕	✕	✕	✕	✕

Δ9-THC, (−)-trans-Δ9-tetrahydrocannabinol; symptoms highlighted in bold were still observable 2.5 h after the injection.

**Table 3:** Psychosis-related general psychopathology.

Study Participant	Drug	Somatic Concern	Anxiety	Guilt Feelings	Tension	Mannerism and Posturing	Depression	Motor Retardation	Uncooperative	Unusual Thought Content	Disorientation	Poor Attention	Lack of Judgment & Insight	Disturbance of Volition	Poor Impulse Control	Preoccupation	Active Social Avoidance
**male 1**	Δ9-THC	✓minimal	✕	✕	✕	✓minimal	↓	✕	✕	✕	**✓minimal**	**✓mild**	✕	✕	✓minimal	**✓mild**	✕
**male 2**	Δ9-THC	✕	✕	✕	✕	✓minimal	✕	✓mild	✕	✕	✓mild	✓mild	✕	✓mild	✕	✓mild	✓mild
**male 3**	Δ9-THC	✓mild	✕	✓ mild	✕	✕	✓minimal	✕	✕	✓mild	**✓mild**	**✓moderate**	✕	✓moderate	✓mild	✓mild	✓minimal
**male 4**	Δ9-THC	✕	✕	✕	✕	✕	✕	✕	✕	✕	✕	✕	✕	✕	✓minimal	✕	✕
**male 5**	Δ9-THC	✓mild	✓mild	✕	✓mild	✕	✓mild	✕	✕	✕	✓mild	✓moderate	✕	✓mild	✓mild	✓mild	✕
**male 6**	Δ9-THC	✕	✕	✕	✕	✓moderate	✕	✕	✕	✓mild	✓minimal	✓mild	✓mild	✓mild	✓mild	✕	✕
**male 7**	Δ9-THC	✓mild	✓minimal	✕	✓minimal	✕	✕	✓mild	✓minimal	✓minimal	**✓mild**	**✓moderate**	✓mild	✓moderate	✕	✕	✕
**female 1**	Δ9-THC	✕	✕	✓ minimal	✓minimal	✕	✓minimal	✓mild	✓mild	✓minimal	✓minimal	✓mild	✕	✓mild	✓mild	✕	✓minimal
**female 2**	Δ9-THC	✕	✕	✕	✕	✕	✕	✓minimal	✕	✕	✕	✕	✕	✕	✓minimal	✕	✕
**female 3**	Δ9-THC	✓mild	✕	✕	✓mild	✕	✕	✕	✕	✕	✕	✓mild	✕	✓mild	✕	✓mild	✕
**female 4**	Δ9-THC	✕	✕	✕	✕	✓minimal	✕	✓mild	✓minimal	✓mild	✓mild	✓moderate	✓mild	✓mild	✓mild	✕	✓minimal
**female 5**	Δ9-THC	**✓mild**	**✓moderate**	✕	**✓mild**	✓minimal	✓minimal	✕	✕	✓mild	✓mild	✓mild	✕	✓mild	✕	✓moderate	✕
**female 6**	Δ9-THC	✕	✕	✕	✕	✓mild	✓mild	✓moderate	✕	✕	**✓mild**	✓moderate	✓minimal	✓mild	✕	✕	✓mild
**female 7**	Δ9-THC	✓mild	✓mild	✕	✓mild	✕	✕	**✓mild**	✕	✕	✕	**✓mild**	✕	✓mild	✓minimal	✓minimal	✓mild
**female 8**	Δ9-THC	✓mild	✓mild	✕	✓mild	✓mild	✕	**✓mod./severe**	**✓minimal**	✓moderate	**✓mild**	**✓moderate**	✓moderate	✓moderate	✕	✓mod./severe	✓mild
**female 9**	Δ9-THC	✕	✕	✕	✕	✓minimal	✕	✕	✓mild	✓mild	✕	✓moderate	✓mild	✕	✓minimal	✕	✕
**male 1**	placebo	✕	✕	✕	✕	✕	✕	✕	✕	✕	✕	✕	✕	✕	✕	✕	✕
**male 2**	placebo	✕	✕	✕	✕	✕	✕	✕	✕	✕	✕	✕	✕	✕	✕	✕	✕
**male 3**	placebo	✕	✓minimal	✕	✕	✕	✕	✕	✕	✕	✓minimal	✕	✕	✕	✕	✕	✕
**male 4**	placebo	✕	✕	✕	✕	✕	✕	✕	✕	✕	✕	✕	✕	✕	✕	✕	✕
**male 5**	placebo	✕	✕	✕	✕	✕	✕	✕	✕	✕	✓mild	✓mild	✕	✕	✓mild	✕	✕
**male 6**	placebo	✕	✓minimal	✕	✓minimal	✕	✕	✕	✕	✕	✕	✕	✕	✕	✕	✕	✕
**male 7**	placebo	✕	✕	✕	✕	✕	✕	✕	✕	✕	✕	✓mild	✕	**✓mild**	✕	✕	✕
**female 1**	placebo	✓mild	✓minimal	✕	✓mild	✓minimal	✕	✕	✕	✕	✕	✕	✕	✕	✕	✓mild	✕
**female 2**	placebo	✕	✕	✕	✕	✕	✕	✕	✕	✕	✕	✕	✕	✕	✕	✕	✕
**female 3**	placebo	✕	✕	✕	✕	✕	✕	✕	✕	✕	✕	✓minimal	✕	✕	✕	✕	✕
**female 4**	placebo	✕	✕	✕	✕	✕	✕	✕	✕	✕	✕	✕	✕	✕	✕	✕	✕
**female 5**	placebo	✕	✕	✕	✕	✕	✕	✕	✕	✕	✕	✕	✕	✕	✕	✕	✕
**female 6**	placebo	✕	✕	✕	✕	✕	✕	✕	✕	✕	✕	✕	✕	✕	✕	✕	✕
**female 7**	placebo	✕	✕	✕	✕	✕	✕	✕	✕	✕	✕	✕	✕	✕	✕	✕	✕
**female 8**	placebo	✕	✕	✕	✕	✕	✕	✕	✕	✕	✕	✕	✕	✕	✕	✕	✕
**female 9**	placebo	✕	✕	✕	✕	✕	✕	✕	✕	✕	✕	✕	✕	✕	✕	✕	✕

Δ9-THC, (−)-trans-Δ9-tetrahydrocannabinol; symptoms highlighted in bold were still observable 2.5 h after the injection.

**Table 4:** Subjects’ quotes.

**Study Participant**	**Δ9-THC**	**Symptom**
male 1	‘I was so stressed, irritable, and prone to anger that I started an argument with my partner the afternoon after the study’	Hostility, irritability, mood lability
male 2	‘I feel I am all over the place and can’t stop laughing, thinking you will expose me, I will say something stupid or strange’	Conceptual disorganization, thought disorder, loosening of associations, excitement
male 3	‘I can’t follow my thoughts, I am not able to think’	Conceptual disorganization, racing thoughts
male 3	‘I can understand things better and look for details, I am superior to others’	Grandiosity
male 3	‘I might have done something wrong, not willing to say’	Feelings of guilt
male 3	‘I am thinking about death and cemeteries’	Unusual thought content
male 6	‘The injection changed me into someone with increased abilities’	Grandiosity
male 7	‘I am feeling quite confused and disoriented, like time is passing slower and the space is different, like from a camera zoom’	Conceptual disorganization, disorientation
female 1	‘I am not interested and I am not willing to talk, I don’t care, I want just to go... I feel down, under the weather; maybe I am depressed’	Negative symptoms, depression
female 4	‘What have you done to me? I understand, you want to make me paranoid with brainwashing questions...’	Suspiciousness/paranoia with loss of insight
female 4	‘What an apple and a ball have in common...You can eat the apple, but not the ball’	Difficulty in abstract thinking
female 5	‘I thought you were going to attack me, people are entering the room to check on me’	Suspiciousness/paranoia, ideas of reference
female 7	‘What an apple and a ball have in common...You can put the apple in the ball’	Difficulty in abstract thinking
female 8	‘My mind went blank, empty, with no thoughts’	Thought blocking
female 8	‘The ventilator’s noise is louder...This noise is actually rain, it’s raining inside the room, I can see and feel it, there is a black sky with seven blue drops, I can count them, someone has opened the ceiling to let the rain in, and put my bed closer to the ceiling...Maybe someone is projecting a sky in front of me’	Inability to ‘filter’ out irrelevant background stimuli, hallucinatory behavior, secondary delusions
female 9	‘Is this real? Is this a fake interview made by a fake doctor, like a Truman show?’	Depersonalization/derealization
female 9	‘Colors are brighter, noises louder, and I have something making a noise in the back of my head’	Hallucinatory behavior
male 3, 7; female 5, 7	‘I think I am going to chock up...something is wrong with my body...the heart is racing’	Preoccupation/somatic concern, anxiety/tension
**Study Participant**	**Placebo**	**Symptom**
male 7	‘I felt spaced out, a little bit paranoid and upset after a conversation with someone who had a strange facial expression’	Suspiciousness/paranoia

Δ9-THC, (−)-trans-Δ9-tetrahydrocannabinol.

**Table 5:** Additional symptoms.

**Study Participant**	**Δ9-THC**
	*physical reactions*
female 2	generalized tremors, vagal reaction
female 3	about to faint
female 5	about to faint, paleness
female 6	about to faint, orthostatic hypotension, sick feeling
female 8	flushing
	*observed symptoms*
males 2, 3, 6; females 1, 2, 3, 4, 8	Different handwriting, errors and corrections in filling out the questionnaires (still present at 2.5 h after the injection for males 3 and 6)
male 6	The participant kept the arm in a distinctively awkward position for 30 min
female 4	The participant committed errors when asked four times to recall words related to a memory task (night instead of black 2/4, vegetable instead of apple 3/4, crisis instead of cries 4/4)
female 8	The participant was internally absorbed and didn’t engage at all with a cognitive task
	*reported long-lasting symptoms (telephone follow-up)*
male 1	suspiciousness, hostility, tension, and poor impulse control until afternoon
male 1	headache, sick and weak feeling, fatigue, exhaustion, physical and mental strain until day after
male 2	tiredness, sleepiness, postprandial somnolence
male 6	disorientation and tiredness until evening
female 5	tiredness and cravings for savory foods until afternoon
female 6	tiredness, sleepiness, and hunger until the end of the day
female 9	sleepiness, thirst, and hungry
females 1 and 4	Symptoms reported during the trial were the same experienced in the past when using recreational cannabis
**Study participant**	**Placebo**
	*physical reactions*
female 1	flushing, drowsiness, stomach pain
female 6	flushing, increase in heart rate, heavy chest feeling
	*reported long-lasting symptoms (telephone follow-up)*
male 7	suspiciousness until afternoon

Δ9-THC, (−)-trans-Δ9-tetrahydrocannabinol.
